# Osteomyelitis and Septic Arthritis in the Darwin Prospective Melioidosis Study

**DOI:** 10.1093/ofid/ofae741

**Published:** 2024-12-19

**Authors:** Stuart Campbell, Dane Hicks, Rajendra P Shetty, Bart J Currie

**Affiliations:** Department of Medicine, Royal Darwin Hospital, Darwin, Northern Territory, Australia; Department of Surgery, Royal Darwin Hospital, Darwin, Northern Territory, Australia; Department of Surgery, Royal Darwin Hospital, Darwin, Northern Territory, Australia; Department of Medicine, Royal Darwin Hospital, Darwin, Northern Territory, Australia; Menzies School of Health Research, Charles Darwin University, Darwin, Northern Territory, Australia

**Keywords:** bone and joint infection, *Burkholderia pseudomallei*, melioidosis, osteomyelitis, septic arthritis

## Abstract

**Background:**

Melioidosis is a multisystem infectious disease caused by the environmental bacterium *Burkholderia pseudomallei*. Osteomyelitis (OM) and septic arthritis (SA) are uncommon primary presentations for melioidosis but important secondary foci, often requiring prolonged therapy and multiple surgeries. We characterized the epidemiology, presentation, treatment, and outcomes of patients from 24 years of the Darwin Prospective Melioidosis Study (DPMS).

**Methods:**

DPMS patients from October 1, 1999, until September 30, 2023, were included if they had a primary or secondary diagnosis of OM or SA. Epidemiological, risk factor, clinical, and outcome data were retrieved from the DPMS database. Antibiotic and surgical data were collated from patient records.

**Results:**

From 1129 consecutive patients with culture-confirmed melioidosis, 122 (10.8%) had OM and/or SA, with 115 evaluable. Ninety-four of 1129 (8.3%) had OM, and 62/1129 (5.5%) had SA, with 41/115 (35.7%) of these having both OM and SA. Many combined infections involved contiguous bone and joints or soft tissue. Fifty-nine (51.3%) were male, and only 4.3% were ≤16 years old. Diabetes mellitus was present in 69.6%, and only 12.2% had no identifiable clinical risk factor. There were 8 deaths (7.0%) and 20 (17.4%) recurrent infections. Seventy-one (61.7%) had operative management, with combined infection associated with more procedures and longer length of stay.

**Conclusions:**

The current paradigm of care for osteoarticular melioidosis involves prolonged intravenous antibiotics in conjunction with timely and complete operative management, and in our setting where these are available, outcomes are good. In many melioidosis-endemic regions these resources are limited, and mortality remains high.

Melioidosis is an opportunistic and often systemic infection increasingly recognized in tropical and subtropical locations globally [1]. The causative sapronotic organism *Burkholderia pseudomallei* has an environmental niche in soil and water, with infection commonly following percutaneous inoculation. Inhalation of aerosolized *B. pseudomallei* can occur during severe weather events such as cyclones, hurricanes, and typhoons, as can ingestion of *B. pseudomallei* from drinking contaminated untreated water [1].

The most common infectious syndrome associated with melioidosis is pneumonia, accounting for over half of all presentations. Melioidosis is associated with diverse involvement of many organ systems including genitourinary, skin and soft tissue, and central nervous systems [2, 3]. Osteomyelitis (OM) and septic arthritis (SA) are uncommon as primary presentations but are increasingly recognized as secondary foci, and treatment entails prolonged therapy and often multiple surgeries, especially for OM [4–6].

The presentation and management of the osteoarticular manifestations of melioidosis have been described previously in modest cohorts from Northern Australia, Thailand, Malaysia, and India [4–8]. These have reported variable incidences, risk factors, surgical and medical treatment modalities, and outcomes, with the most consistent finding being the link between diabetes mellitus (DM) and osteoarticular melioidosis compared with other presenting melioidosis syndromes.

The Darwin Prospective Melioidosis Study (DPMS) is a longitudinal cohort study that began in 1989, based at Royal Darwin Hospital and the Menzies School of Health Research [3]. We aimed to characterize the clinical presentation, management, and outcomes of OM and SA in the DPMS to assess and refine the antibiotic and surgical guidelines that have evolved from earlier analyses [1, 5, 9].

## METHODS

The DPMS documents and manages all patients with culture-confirmed melioidosis in the tropical Top End of the Northern Territory of Australia, as previously described [3]. Patient demographic, epidemiological, clinical, and laboratory details are stored prospectively in MariaDB, version 10.2.31 (Oracle, CA, USA). Each patient is assigned a single primary clinical syndrome diagnosis on presentation, and secondary clinical syndromes (if they occur) that subsequently develop are further categorized into acute (symptoms present for <2 months) or chronic (symptoms present for ≥2 months) melioidosis [3]

DPMS patients from the 24 years between October 1, 1999, and September 30, 2023, were included in this study if they had a primary or secondary diagnosis of OM or SA. This time frame included some patients from previous studies, though this analysis presents additional data not collected in these previous analyses [5, 9]. Other data extracted from the DPMS database included age, sex, ethnicity, and geographic region. Clinical risk factors included DM, hazardous alcohol use, chronic kidney disease, chronic lung disease, active malignancy, immunosuppression, and rheumatic heart disease. Recreational and occupational environmental exposure to *B. pseudomallei* were recorded, as were presumptive infecting events (inoculation or inhalation). Antimicrobial therapies and operative management of patients were retrieved retrospectively from the electronic and hard copy patient records.

Antibiotic treatment data were collected for both the intensive (intravenous) and eradication (oral) phases. Typical intravenous backbone therapy is ceftazidime or meropenem, and for eradication therapy trimethoprim/sulfamethoxazole (TMP/SMX) is strongly preferred (Supplementary Table 1) [1]. The antibiotic that was prescribed for the longest duration for each phase was considered the predominant antibiotic of that phase, and complications and their management were extracted from notes. Duration of the intensive phase was determined through the electronic medical records and prescribing software, while the duration of the eradication phase was assessed through manual review of outpatient clinic letters. For those with patient-led discharge or nonadherence to therapy, it was assumed that the duration of eradication therapy would equal the number of days of antibiotics supplied on discharge (typically 2 weeks) unless outpatient documentation reported otherwise. Nonadherence to therapy was determined using the notes and was considered present if there was a gap of >72 hours for intensive phase therapy or 7 days for eradication phase therapy.

Surgical data were collected through review of the operation notes and other patient record entries, including some data previously collected [5]. We collected the site, type of operation, and microbiology results of collected tissue and joint fluid specimens. Operations for OM were grouped into categories of minor (sampling only, debridement, or incision and drainage) and major (cortical debridement, sequestrectomy, reaming, amputation, cement placement, or hardware removal).

Recurrent melioidosis was defined as *B. pseudomallei* culture–positive recurrent clinical findings or radiological progression requiring re-admission after the patient had been discharged from acute inpatient care (though they may still have been “admitted” to the outpatient parenteral antimicrobial therapy service). Recurrent melioidosis is divided into recrudescence, whereby the recurrence occurs during the period of ongoing antibiotic therapy (even if the patient is nonadherent), and relapse or new infection, whereby the recurrence occurs after the scheduled completion of therapy. Bacterial genotyping distinguishes between relapse (initial and recurrent isolates, identical genotypes) and new infection (initial and recurrent isolates, different genotypes).

DPMS and antibiotic and surgical data were collated into a standalone electronic database (RedCAP; Yale University, New Haven, CT, USA) [10, 11]. Statistical analysis was performed using STATA 17 (StataCorp, College Station, TX, USA). Categorical data were analyzed via the Fisher exact method, with further comparisons performed using adjusted residuals and Bonferroni correction if initial testings found *P* < .05. Student *t* tests or the Kruskal-Wallis equality-of-populations rank test (followed by Dunn's pairwise comparison if *P* < .05) were performed on continuous parametric and nonparametric data, respectively. Logistic regression was performed to assess the interaction between normally distributed continuous data and binary categorical outcomes. Annual incidence data were calculated using the estimated 2023 population residing in melioidosis-endemic areas [12].

## RESULTS

Of 1129 patients with culture-confirmed melioidosis over the 24 years from October 1, 1999, to September 30, 2023, 122 (10.8%) had OM and/or SA. Of these, 4 patients were excluded due to unconfirmed osteoarticular involvement, 2 as they were transferred interstate during their acute admission, and 1 as the paper records containing the operative report details had been destroyed, leaving 115 available for analysis. OM was the most common clinical syndrome (94/1129; 8.3%), while SA comprised 62/1129 (5.5%) episodes. Both OM and SA (“combined infection”) occurred in 41/115 (35.7%). The annual incidence of osteoarticular melioidosis was 2.5/100 000, with the number of cases ranging from 1 to 17 per season (Supplementary Figure 1).

Of the 53 OM-only infections, 17/53 (32.1%) had OM as the presenting melioidosis infection, and 36/53 (67.9%) had OM develop secondary to another clinical diagnosis. Of the 21 SA-only infections, 10/21 (47.6%) had SA as the presenting melioidosis infection, and 11/21 (53.4%) had SA develop secondary to another clinical diagnosis. The presenting syndrome for combined infections was SA in 19/41 (46.3%) and OM in 2/41 (4.9%). The nonosteoarticular presenting syndrome was most frequently pneumonia in all 3 of OM-only (12/53 [22.6%]), SA-only (6/21 [28.6%]), and combined infections (15/41 [36.6%]). Of the 115 cases, 9 (7.8%) did not have OM or SA during their initial admission with melioidosis but were first diagnosed when admitted with recurrent melioidosis (7 recrudescent and 2 relapsed). Patients typically had 2 or more clinical syndromes diagnosed during their admission; only 12/115 (10.4%) had a single infective syndrome. Notably, soft tissue abscesses were seen significantly more frequently in OM-only (16/53; 30.2%) compared with SA-only (2/21; 9.5%) or combined infections (3/41; 7.3%; *P* < .01). A site of OM was underlying or associated with the focus of soft tissue infection in 14/16 (87.5%) of these patients.

Demographic and risk factor data are displayed in Table 1, stratified by focus of infection (OM-only, SA-only, or combined). Of the 115 patients, 59 (51.3%) were male, and the mean age (SD) was 48.7 (14.9) years. Only 5 (4.3%) were children age ≤16 years (range, 9 months to 15 years); 1 had DM, but the others had no identifiable risk factor. Most (81/115; 70.4%) patients were Aboriginal and/or Torres Strait Islander. The majority were geographically located in the greater Darwin region (79/115; 68.7%). The most common risk factor was DM, present in 80/115 (69.6%) patients. The median number of clinical risk factors (interquartile range [IQR]) was 1 (1–2), and 16/115 (13.9%) patients had no identifiable clinical risk factor. The number of risk factors present was not associated with clinical syndrome, recurrence, or death (*P* = .52, *P* = .31, and *P* = .66, respectively). Occupational and recreational exposure to environmental *B. pseudomallei* were common (Table 1), as previously defined and described for DPMS patients (Supplementary Table 1 in [3]). Infection attributed to prior specific putative infecting events occurred in 20/115 (17.4%) patients. Eight of these were a specific event where percutaneous inoculation likely occurred, with subsequent osteoarticular melioidosis in an adjacent joint and/or bone, and often also involving local soft tissues. Examples included a 7-year-old who fell onto his elbow 3 days before presentation with elbow SA and distal humerus OM and a 60-year-old who sustained a compound fractured ankle and developed distal tibial osteomyelitis. Melioidosis bacteremia was present in 82/115 (71.3%) patients; specifically, 33/53 (62.3%) in OM-only, 16/21 (76.2%) in SA-only, and 33/41 (80.5%) in those with combined infection. Bacteremia was not associated with recurrence or death (*P* = .79 and *P* = 1.0, respectively).

**Table 1.** Demographic Factors, Risk Factors, Clinical Factors, Therapeutic Data, and Outcomes of Patients Admitted With Osteoarticular Melioidosis in the Darwin Prospective Melioidosis Study Stratified by Bone and/or Joint Involvement

…			Osteomyelitis Only (n = 53)	Septic Arthritis Only (n = 21)	Combined (n = 41)	*P* Value
Demographic	Age, No. (SD)		48.11 (12.89)	48.52 (19.05)	49.61 (15.40)	.89
	Male sex, No. (%)		26 (49.1)	14 (66.7)	19 (46.3)	.29
	Aboriginal and/or Torres Strait Islander ethnicity, No. (%)		40 (75.5)	13 (61.9)	28 (68.3)	.48
Risk factors	Diabetes mellitus, No. (%)		35 (66.0)	12 (57.1)	33 (80.5)	.13
	Hazardous alcohol use, No. (%)		21 (39.6)	7 (33.3)	15 (36.6)	.91
	Chronic kidney disease, No. (%)		8 (15.1)	4 (19.0)	4 (9.8)	.57
	Chronic lung disease, No. (%)		3 (5.7)	4 (19.0)	10 (24.4)	.03
	Malignancy, No. (%)		3 (5.7)	1 (4.8)	3 (7.3)	.91
	Immunosuppressed, No. (%)		3 (5.7)	2 (9.5)	1 (2.4)	.48
	RHD, No. (%)		5 (9.4)	2 (9.5)	6 (14.6)	.70
	Previous melioidosis		4 (7.5)	0 (0.0)	5 (12.2)	.31
Clinical factor	Concomitant syndrome, No. (%)	BNF	4 (7.5)	0 (0.0)	1 (2.4)	.27
		Genitourinary	4 (7.5)	3 (14.3)	6 (14.6)	.50
		Pneumonia	25 (47.2)	15 (71.4)	23 (56.1)	.16
		Skin abscess	5 (9.4)	2 (9.5)	6 (14.6)	.70
		Soft tissue Abscess	16 (30.2)	2 (9.5)	3 (7.3)	<.01
		CNS	4 (7.5)	2 (9.5)	1 (2.4)	.45
	Occupational exposure		5 (9.4)	3 (14.3)	8 (19.5)	.37
	Recreational exposure		48 (90.6)	16 (76.2)	35 (85.4)	.27
	Documented bacteremia		33 (62.3)	16 (76.2)	33 (80.5)	.14
	Shock		9 (17)	5 (24)	13 (32)	.25
	ICU admission		15 (28)	9 (43)	14 (34)	.48
	Long bone involvement		23 (43.4)	NA	32 (78.1)	<.01
Therapeutic information	Managed operatively		19 (36)	17 (81)	35 (85)	<.01
	Operations required^a^ (95% CI)		2.2 (1.4–2.8)	1.8 (1.3–2.1)	3.4 (2.7–4.1)	<.01
	IV antibiotic duration (95% CI), d		51.3 (46.3–56.3)	44.4 (33.5–55.4)	56.3 (47.5–65.1)	.16
	PO antibiotic duration (95% CI), d		155.2 (131.4–179.1)	155.4 (112.5–198.4)	159.5 (132.4–186.6)	.99
	IV therapy nonadherence		3 (5.6)	0 (0)	4 (9.8)	.35
	PO therapy nonadherence		18 (35)	5 (28)	14 (36)	.82
Outcome	Recurrent melioidosis	Recrudescence	10 (19)	1 (5)	7 (17)	1.0
		Relapse	1 (2)	0 (0)	1 (2)	-
	Median length of stay (IQR), d		22 (14–30)	26 (16–46)	32 (22–48)	.02
	Died	Acute	2 (4)	3 (14)	2 (5)	1.0
		Relapse	0 (0)	0 (0)	1 (2)	-

Abbreviations: BNF, bacteremia no focus; CNS, neurological melioidosis; ICU, intensive care unit; IV, intravenous; PO, per oral; IQR, interquartile range, RHD, rheumatic heart disease.

^a^Patients managed nonoperatively were excluded from this analysis.

**Figure 1.**
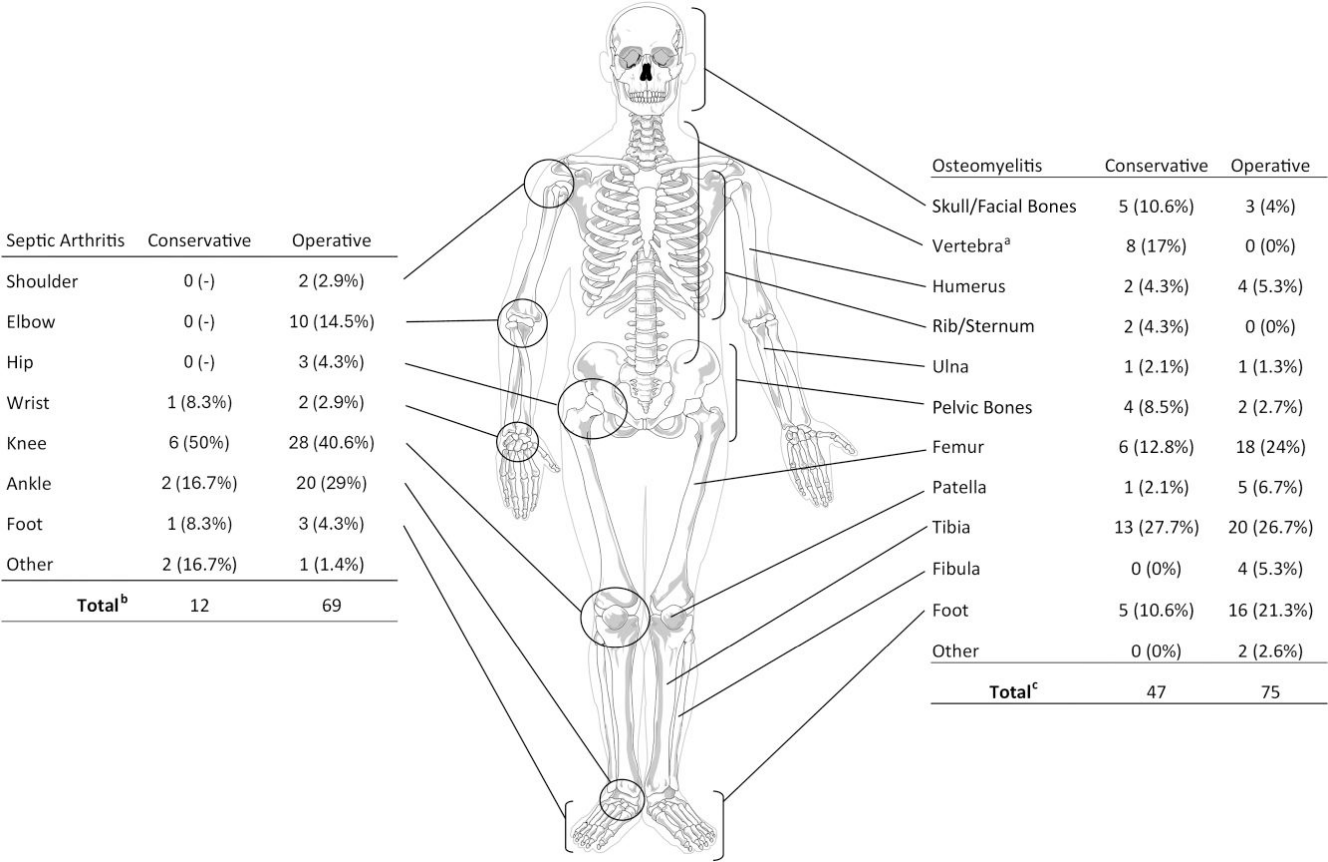
Diagram of the cumulative total foci of infection (both septic arthritis and osteomyelitis) for 115 patients with osteoarticular melioidosis in the Darwin Prospective Melioidosis Study (DPMS). ^a^Represents foci with significant differences between groups. ^b^Seven patients had 2 involved joints, and 6 had 3 involved joints. ^c^Twelve patients had 2 osteomyelitis foci, and 8 had 3 foci.

Infection involved many different joint and bone foci, with some patients having multiple sites involved. The 94 patients with OM had 122 total sites of bone infection, while the 62 patients with SA had 81 total joints involved (Figure 1). Seven patients had 2 involved joints, and 6 had 3 involved joints, while 12 patients had 2 OM foci, and 8 had 3 foci. The most common site of OM was the tibia (33/122; 27%), followed by the femur (24/122; 19.7%). SA was most often seen in the lower limb, with the knee (34/81; 42%) and ankle (22/81; 27.2%) most represented. Long bone involvement was seen in 23/53 (43.4%) OM-only patients, but in 32/41 (78.1%) combined infections (*P* < .01). Rates of operative and nonoperative management differed significantly between foci of OM (*P* < .01), though not between foci of SA (*P* = .10). This association was driven by the complete (8/8; 100%) conservative (nonsurgical) management of vertebral OM (*P* < .01). Of note, there was no case of prosthetic joint melioidosis SA and only a single case of melioidosis OM involving metalware; in a 56-year-old with a thigh wound infection communicating with preexisting femur hardware, the infection presumptively followed wound exposure to environmental *B. pseudomallei*.

Seventy-one (of 115; 61.7%) patients underwent operative management of either their OM, SA, or both (Table 1; Supplementary Table 2). OM-only presentations were managed operatively less frequently then either SA or combined syndromes (*P* < .01), while combined infections required significantly more procedures (median [IQR], 3 [2–5]) than either OM- (median [IQR], 2 [1–3]; *P* < .01) or SA-only (median 2, IQR 1–2; *P* < .01). Patients undergoing operative management for OM typically had major procedures performed initially, followed by 1 or more minor procedures, while operatively managed SA patients usually underwent arthroscopy before progressing to arthrotomy (Table 2), though both these trends were nonsignificant (*P* = .07 and .10, respectively). There were differences in median length of stay (LOS) between infection foci, which was driven by an increased LOS in combined infections compared with OM-only (*P* < .01).

**Table 2.** Type of Procedure Performed Stratified by Sequential Operations

	Operation	1st, No. (%)	2nd, No. (%)	3rd, No. (%)	4th, No. (%)	5th, No. (%)	*P* Value (for Trend^a^)
OM	Minor^b^	20 (66.6)	16 (72.7)	7 (70)	5 (71.4)	4 (100)	…
	Major^b^	16 (44.4)	6 (27.3)	3 (30)	2 (28.6)	0 (0)	…
	Total	36	22	10	7	4	.07
SA	Arthroscopy	20 (40)	14 (36.8)	6 (27.3)	3 (33.3)	1 (11.1)	…
	Arthrotomy	30 (60)	24 (61.2)	16 (72.7)	6 (66.7)	8 (88.9)	…
	Total	50	38	22	9	9	.10

Abbreviations: OM, osteomyelitis; SA, septic arthritis.

^a^ *P* value calculated using the chi-square statistic for trend.

^b^Operations considered minor were sampling only, debridement, or incision and drainage, while all other operations (cortical debridement, sequestrectomy, reaming, amputation, cement placement, and hardware removal) were considered major.

Eight (of 115; 7%) died from melioidosis, the majority (7/8; 87.5%) in the acute phase of their illness, and 2/8 (25%) ≤5 days after initial admission. Four patients died in intensive care from sepsis, 2 elderly patients were palliated after family consultation, and 2 had progressive malignancies. Death was not associated with any specific presenting syndrome (*P* = .73) or operative strategy (*P* = 1.0).

There were 20/115 (17.4%) who had OM and/or SA who subsequently had recurrent melioidosis, 18 recrudescences with failure of therapy, and 2 relapsed melioidosis post–completion of therapy (Table 1). The median time to recurrent melioidosis (IQR) was 63.5 (35.5–129.5) days from acute inpatient discharge. There were no associations between recurrence and anatomical site of infection in either OM or SA (*P* = .36 and *P* = .23, respectively). Of the 20 recurrent melioidosis admissions, 4 lacked residual osteoarticular involvement (2 re-presented with pneumonia, 1 with neurological melioidosis, another unspecified). Of the 16 others, 13 had OM and 3 had combined OM and SA on recurrence. All except 1 (15/16; 81.3%) OM recurrence location were at the same site, and all SA was in the same joint as affected initially.

**Figure 2.**
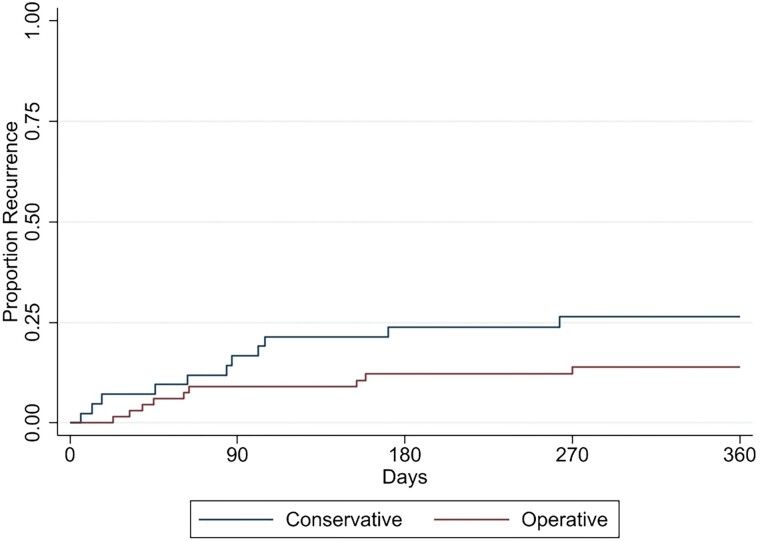
Kaplan-Meier failure curve comparing the proportion of patients with recurrent melioidosis over time since discharge from acute admission between conservative (nonoperative) or operative management strategies.

An association between operative strategy and recurrence was explored (Figure 2). Nine (of 71; 12.7%) patients managed operatively had a recurrence of melioidosis compared with 11/44 (25%) of those managed conservatively (*P* = .09) (Supplementary Table 2). In OM managed operatively, a major procedure initially was not associated with reduced recurrence (*P* = .67) or LOS (*P* = .84); there was a trend toward an increased number of total procedures (*P* = .08). Up-front arthrotomy over arthroscopy was not associated with reduced procedures, different LOS, or recurrence (*P* = .49, *P* = .57, and *P* = .46 respectively).

There was a significant association between intravenous therapy phase nonadherence and recurrent infection; 4/7 (57.1%) with nonadherence had recurrent infection (*P* = .02) (Supplementary Table 3). Though the median total duration of intravenous therapy was significantly lower in those with nonadherence (28.3 days; 95% CI, 6.8–49.7; vs 53.4 days; 95% CI, 49.1–57.7; *P* < .01), logistic regression did not find an association between total length of intravenous therapy and recurrence. An association between oral eradication therapy nonadherence and recurrence was not seen (*P* = .17).

There was no association between long bone OM and operative strategy, recurrence, or death (*P* = .15, *P* = .33, and *P* = 1.0 respectively). However, in patients who were managed operatively, those with long bone involvement required significantly more median (IQR) operating theater attendances (3 [2–5]) than those without (2 [1–2]; *P* < .01). We investigated long bone OM foci and proximity to affected joints in patients with combined infections where the OM involved long bones (Table 3); there were 36 episodes of SA and long bone OM among 32 patients, the majority having OM contiguous with the affected joint (32/36; 88.9%). The involved joint–bone foci for the other 9 combined infections commonly involved feet and toes and are detailed in Supplementary Table 4. SA of the knee was most associated with contiguous OM—12/36 (33.3%) had knee SA and distal femur OM, and 9/36 (25%) had knee SA and proximal tibia OM.

**Table 3.** Site of Osteomyelitis (Contiguous or Noncontiguous) Stratified by Involved Joint

		Site of Osteomyelitis, No. (%)	
Involved Joint	Involved Bone	Contiguous	Noncontiguous
Knee	Femur	12 (37.5)	0 (0)
	Tibia	9 (28.1)	2 (6.3)
Ankle	Tibia	3 (9.4)	1 (3.1)
	Fibula	2 (6.3)	0 (0)
Hip	Femur	1 (3.1)	1 (3.1)
Elbow	Humerus	3 (9.4)	0 (0)
	Ulna	1 (3.1)	0 (0)
Shoulder	Humerus	1 (3.1)	0 (0)
…	Total	32 (88.9)	4 (11.1)

Ceftazidime was the most common intravenous phase antibiotic used (in 81/115; 70.4%), while TMP/SMX was the most common eradication phase therapy (in 73/115; 63.5%). After exclusion of 1 patient who died on day 3 of presentation and who was treated with meropenem, there were no significant differences between the choice of therapy and admission duration, recurrence, or death for any intravenous or oral phase therapy (Supplementary Tables 5 and 6).

There were 19 documented operative complications, most commonly bleeding requiring either repeat operating theater attendance or blood products (6/19; 31.6%) or wound infection/breakdown (6/19; 31.6%). Only a single pathological fracture was identified. Intravenous antibiotics were generally well tolerated, with 10/115 (8.7%) patients having an adverse drug reaction and 9 requiring cessation of ceftazidime (Supplementary Table 7). Oral therapy was associated with more adverse drug reactions (32/115; 27.8%), predominately due to TMP/SMX (22/32; 68.8%), which was ceased in 17/22 (77.2%) cases (Supplementary Table 7). The most common adverse effect was cytopenia (8/22; 36.2%), with hepatitis and renal dysfunction each affecting 3/22 (13.6%).

## DISCUSSION

Overall, 10.8% of patients in the DPMS had OM and/or SA. While combined OM and SA were seen in 35.7% of these, OM was more common. Both OM and SA can be the presenting clinical syndrome for melioidosis, but it is more common for OM and/or SA to develop secondary to admission with another primary focus of melioidosis, typically pneumonia. The incidence of osteoarticular melioidosis over the study period was 2.5/100 000 per year, which is approximately a third of the underlying rate of bone and joint infections described in our institution previously [13, 14]. There was, however, substantial season-to-season variation seen, consistent with previous reports from the DPMS [3].

Diabetes mellitus is the most important and common clinical risk factor for melioidosis at any site [1]. Our data suggest that this association is even stronger for those with osteoarticular melioidosis, as 69.6% of patients in this cohort have DM compared with the baseline rate of 45% in the DPMS, which is consistent with other studies (79%–88%) [3, 4, 15, 16]. A lack of risk factors appears protective against osteoarticular melioidosis, as only 13.9% in our study lacked an identifiable risk factor, again comparable to previous data (2.3%–5.1%) [4, 15, 16]. Children also are seldom afflicted by melioidosis OM or SA; prior studies that included children did not identify any cases, and in the current study only 4.3% were ≤16 years old [4, 16]. Interestingly, we found only a single case of infection involving prosthetic material, similar to previous reports of osteoarticular melioidosis, which stands in contrast to bone and joint infections caused by other pathogens whereby the presence of foreign material is a risk factor for infection [4, 17].

We found a melioidosis recurrence rate of 17.4% in this study, with 18/20 being recrudescent melioidosis during the therapeutic phase and only 2 being bacterial genomically confirmed relapse after completion of therapy. While this relapse rate in OM and SA melioidosis was similar to the relapse rate of 3.7% seen in the DPMS overall [3], recrudescence during therapy for OM and SA melioidosis was considered higher than that seen for other melioidosis clinical presentations, although recrudescence during therapy has not been formally prospectively documented in DPMS to confirm this observation. The high recrudescence rate was associated with poor patient adherence to intravenous therapy, and this supports the DPMS guidelines directing longer duration of intravenous therapy for especially melioidosis OA, as well as the need to focus on infection source control with surgery where required. Bone penetration of both intravenous and oral choices for melioidosis therapy is likely to exceed *B. pseudomallei* minimum inhibitory concentrations in bone [18, 19].

These findings are consistent with the concept of melioidosis as an opportunistic infection, whereby healthy people rarely develop severe disease, and with prompt diagnosis and management (including intensive care), death is uncommon and restricted to those with identified clinical risk factors [1, 3]. The overall mortality in our study (7%) is consistent with that seen in north Queensland (3%), a similarly resourced setting with available—and melioidosis-experienced—medical and surgical services [4]. In contrast, studies from China and Thailand have found much higher mortality rates of 20% and 34%, respectively, and this may be related to a limited-resource setting [15, 16]. The restriction of osteoarticular melioidosis to those with clinical risk factors contrasts with bone and joint infections from other common bacterial pathogens, which are seen more routinely in children and healthy adults [13, 15, 20].

Bacteremia was present in 71.3% of DPMS patients with melioidosis OM/SA, compared with 79% in Queensland, 93% in Chinese, and 86% in Thai cohorts [4, 15, 16]. This supports bacteremic spread to bone and/or joint as the primary pathogenetic process in both primary and secondary infections, with the recognition that in combined infections, contiguous spread from joint to bone to neighboring soft tissues (or vice versa) also occurs. There were a small number of patients with specific percutaneous inoculating events who developed subsequent melioidosis SA and/or OM in the underlying structures, often also with concomitant soft tissue and muscle involvement. OM due to melioidosis, like SA, typically affected the lower limb, with the femur and tibia being the most involved sites in this study (27% and 20%, respectively). This lower limb predominance conforms with previous data from both melioidosis-specific and all-cause infections [4, 13, 16]. The high rates of DM and the resultant distal neurovascular damage have been hypothesized to be a cause of this lower limb predilection [4].

When associated with SA, OM was typically contiguous with the involved joint, consistent with local spread rather than hematogenous dissemination. Indeed, the association between SA of the knee and OM of the contiguous femur or tibia warrants routine imaging of these foci for exclusion of concomitant involvement.

We also identified an association between the clinical syndromes of OM and soft tissue abscesses, with nearly 90% of abscesses in those with concomitant OM communicating with the underlying focus of OM. Clinicians thus should suspect possible occult bone involvement local to melioidosis soft tissue abscesses, especially as bone involvement has major management implications for durations of intravenous and oral therapy.

The importance of adequate surgical management of both melioidosis SA and OM has been previously stressed [4–6]. Operative management of osteoarticular melioidosis halved the rate of recurrent melioidosis (12.7% vs 25%; *P* = .09) (Figure 2) in this study but was performed much less frequently in patients with OM compared with either SA alone or combined with OM. Extensive debridement of involved bony foci increases the risk of operative complication or loss of function and, depending on the site, may be technically difficult. Of importance, and in contrast to those with long bone OM, none of those with vertebral melioidosis OM received operative management, with no recurrence in this subgroup.

Arthroscopy is preferred to arthrotomy in nonmelioidosis SA, though advantages of arthrotomy include complete access to both the joint and adjacent bones, which may be important in melioidosis [21]. However, we did not identify any difference in outcomes between up-front arthroscopy and arthrotomy in this cohort. When managed operatively, those with combined OM and SA required significantly more procedures than either SA or OM alone, reflecting a greater local disease burden and the requirement for more intervention for adequate source control. The longer LOS in combined infection compared with OM-only is consistent with the increased severity and complexity of disease.

In addition to adequate surgical source control, antibiotic regimens and durations for osteoarticular melioidosis have evolved following the seminal study from Thailand showing that intravenous ceftazidime halved mortality [1, 3, 5, 22–24]. Our regimen includes a minimum of 4 weeks of intravenous therapy (usually ceftazidime) for SA and 6 weeks for OM, with longer durations required if further surgery occurs for source control, followed by oral eradication therapy for 3 (for SA) and 6 months (in OM) [1, 22]. As expected, our data showed that nonadherence to intravenous therapy was associated with recurrent melioidosis. A similar approach to surgery and antimicrobial treatment in a Queensland cohort was associated with low mortality [4].

Complications of antibiotic therapy were most often seen with oral eradication treatment and were typically due to TMP/SMX. This frequently results in dose reduction of TMP/SMX or cessation and a change to mostly doxycycline or occasionally amoxycillin/clavulanate [4, 22]. These risks of prolonged high-dose TMP/SMX are being addressed through an ongoing prospective randomized trial investigating intravenous-only regimens in Queensland (Australian New Zealand Clinical Trials Registry #ACTRN12622000225718) [25].

This study has several limitations, the most significant being the retrospective collection of surgical data and antibiotic choices and durations for DPMS patients. In addition, over the 24 years of the study the management, operative decision-making, and both surgical techniques and antimicrobial regimens have evolved. Protocols for patient follow-up and a shift to outpatient parenteral antimicrobial therapy–based models of care have changed over this period. Our analysis did not address these longitudinal changes. There is also considerable heterogeneity in the anatomical foci involved, especially for OM, making it difficult to assess the outcomes of specific foci of infection in more granular detail. Another limitation is that we did not collect data on longer-term orthopedic functional outcomes for joints and bones for these patients.

In summary, osteoarticular melioidosis is mostly restricted to patients with clinical risk factors and especially DM. The current care paradigm in osteoarticular melioidosis revolves around timely and adequate source control followed by 4–6 weeks of intravenous antibiotic therapy. This study, and data from similar settings, support this stratagem for the majority of cases [4]. Melioidosis SA usually requires surgical source control, as does more extensive long bone OM, but vertebral OM can be managed with antibiotics alone.

## Supplementary Material

ofae741_Supplementary_Data

## Supplementary Data

Supplementary materials are available at *Open Forum Infectious Diseases* online. Consisting of data provided by the authors to benefit the reader, the posted materials are not copyedited and are the sole responsibility of the authors, so questions or comments should be addressed to the corresponding author.
