# Supplementary material for: Osteomyelitis and Septic Arthritis in the Darwin Prospective Melioidosis Study
Source: Open Forum Infect Dis. 2024 Dec 19;12(1):ofae741. doi: 10.1093/ofid/ofae741 (PMC11697087; doi:10.1093/ofid/ofae741)
Supplement: ofae741_Supplementary_Data [file ofae741_supplementary_data.docx]

Osteomyelitis and septic arthritis in the Darwin Prospective Melioidosis Study

Supplementary Material

Supplementary Table 1: Recommended duration of intensive (Intravenous) and eradication (oral) phases of antimicrobial therapy in for *Burkholderia pseudomallei* stratified by infectious syndrome. Adapted from the 2024 Darwin Melioidosis Treatment Duration Guideline, and Reference 1 Box 2.

| Antibiotic Duration-Determining Focus | | Minimum intensive phase duration (weeks)^a^ | Eradication phase duration (months)^e^ |
| --- | --- | --- | --- |
| Skin abscess | | 2 | 3 |
| Bacteraemia with no focus | | 2 | 3 |
| Pneumonia | | | |
|  | Unilobar pneumonia without lymphadenopathy^b^ or ICU admission and with negative blood cultures | 2 | 3 |
|  | Multilobar pneumonia without lymphadenopathy^b^ or ICU admission and with negative blood cultures;  Or  Unilobar pneumonia without lymphadenopathy^b^ or ICU admission but with positive blood cultures | 3 | 3 |
|  | Pneumonia with either lymphadenopathy^b^ or ICU admission; or multilobar pneumonia with positive blood cultures | 4 | 3 |
| Deep-seated collection and septic arthritis^c^ | | 4^d^ | 3 |
| Osteomyelitis | | 6^d^ | 6 |
| Central nervous system infection and arterial infections (e.g. mycotic aneurysms) | | 8^d^ | 6 |
| a. Use clinical judgement to guide prolongation of intensive phase if improvement is slow or if blood cultures remain positive at 7 days.  b. Defined as enlargement of any hilar or mediastinal lymph node to greater than 10mm diameter.  c. Defined as abscess anywhere other than skin, lungs, bone, CNS or vasculature.  d. Intensive phase duration is timed from date of most recent drainage of collection (e.g. prostatic abscess) or tissue resection, where culture of the drainage specimen or resected material grew *B. pseudomallei* or where no specimen was sent for culture; clock is not reset if drainage specimen is culture negative.  e. Except in CNS melioidosis, trimethoprim+sulfamethoxazole is introduced as a graded dosing. Cessation of intravenous therapy and commencement of the timed eradication phase is set only once the full planned dose of trimethoprim+sulfamethoxazole is reached.  f. Life-long suppressive antibiotic therapy may be required following vascular prosthetic surgery.  Adapted from: Currie *et al* (2023) The 2024 revised Darwin Melioidosis Treatment Guideline. THE NORTHERN TERRITORY DISEASE CONTROL BULLETIN 30:3–11 https://digitallibrary.health.nt.gov.au/nthealthserver/api/core/bitstreams/88d2f610-e0e5-42c3-9770-a734f874ca98/content | | | |

Supplementary Figure 1. Histogram of the osteoarticular melioidosis cases per season (October through September) over the course of the study.

Supplementary Table 2. Demographic factors, risk factors, clinical factors, therapeutic data, and outcomes of patients admitted with osteoarticular melioidosis in the Darwin Prospective Melioidosis Study stratified by operative strategy.

|  | | | Conservative  N=44 | Operative  N=71 | p-value |
| --- | --- | --- | --- | --- | --- |
| Demographic | Age at Diagnosis | | 49.6 (12.2) | 48.2 (16.4) | 0.62 |
|  | Sex | | 25 (57%) | 34 (48%) | 0.44 |
|  | Aboriginal/Torres Strait Islander Ethnicity | | 31 (70%) | 50 (70%) | 1.00 |
| Risk Factors | Diabetes | | 29 (66%) | 51 (72%) | 0.54 |
|  | Hazardous alcohol use | | 19 (43%) | 24 (34%) | 0.33 |
|  | Chronic Kidney Disease | | 3 (7%) | 13 (18%) | 0.10 |
|  | Chronic Lung Disease | | 4 (9%) | 13 (18%) | 0.28 |
|  | Active Malignancy | | 2 (5%) | 5 (7%) | 0.71 |
|  | Immunosuppressed | | 3 (7%) | 3 (4%) | 0.67 |
|  | Rheumatic Heart Disease / Cardiac Failure | | 5 (11%) | 8 (11%) | 1.00 |
| Clinical Factors | Syndrome | Osteomyelitis | 34 (77%) | 19 (27%) | <0.01 |
|  |  | Septic arthritis | 4 (9%) | 17 (24%) |  |
|  |  | Both | 6 (14%) | 35 (49%) |  |
|  | Concomitant syndrome (n, %) | Bacteremia without focus | 4 (9%) | 1 (1%) | 0.07 |
|  |  | Genitourinary | 6 (14%) | 7 (10%) | 0.56 |
|  |  | Pneumonia | 27 (61%) | 36 (51%) | 0.34 |
|  |  | Skin Abscess | 3 (7%) | 10 (14%) | 0.36 |
|  |  | Soft Tissue Abscess | 7 (16%) | 14 (20%) | 0.80 |
|  |  | Neurological | 4 (9%) | 3 (4%) | 0.43 |
|  | Occupational Exposure | | 8 (18%) | 8 (11%) | 0.41 |
|  | Recreational Exposure | | 41 (93%) | 58 (82%) | 0.10 |
|  | Bacteremia | | 33 (75%) | 49 (69%) | 0.53 |
|  | Admission complicated by Septic Shock? | | 7 (16%) | 20 (28%) | 0.18 |
|  | Required ICU Admission? | | 13 (30%) | 25 (35%) | 0.55 |
|  | Long Bone involvement | | 20 (45%) | 35 (49%) | 0.69 |
| Therapeutic factors | IV Antibiotic duration (days) | | 45.7 (22.4) | 55.7 (23.2) | 0.03 |
|  | PO Antibiotic duration (days) | | 145.5 (77.1) | 163.5 (87.5) | 0.29 |
|  | IV Antibiotic non-compliance | | 5 (11%) | 2 (3%) | 0.10 |
|  | PO Antibiotic non-compliance | | 12 (29%) | 26 (38%) | 0.41 |
| Outcomes | Recurrent melioidosis | | 11 (25%) | 9 (13%) | 0.09 |
|  | Died | Acute Illness | 3 (7%) | 4 (6%) | 1.00 |
|  |  | Recurrence | 0 (0%) | 1 (1%) | - |

Supplementary Table 3: Demographic factors, risk factors, clinical factors, therapeutic data, and outcomes of patients admitted with osteoarticular melioidosis in the Darwin Prospective Melioidosis Study stratified by recurrent melioidosis status.

|  | | | No Recurrence  N=95 | Recurrent infection  N=20 | p-value |
| --- | --- | --- | --- | --- | --- |
| Demographics | Age at Diagnosis | | 49.1 (15.7) | 47.0 (10.9) | 0.56 |
|  | Sex | | 46 (48%) | 13 (65%) | 0.22 |
|  | Aboriginal/Torres Strait Islander Ethnicity | | 65 (68%) | 16 (80%) | 0.42 |
| Risk factors | Diabetes | | 63 (66%) | 17 (85%) | 0.12 |
|  | Hazardous alcohol use | | 32 (34%) | 11 (55%) | 0.08 |
|  | Chronic Kidney Disease | | 15 (16%) | 1 (5%) | 0.30 |
|  | Chronic Lung Disease | | 15 (16%) | 2 (10%) | 0.73 |
|  | Active Malignancy | | 5 (5%) | 2 (10%) | 0.35 |
|  | Immunosuppressed | | 6 (6%) | 0 (0%) | 0.59 |
|  | Rheumatic Heart Disease / Cardiac Failure | | 12 (13%) | 1 (5%) | 0.46 |
| Clinical factors | Syndrome | Osteomyelitis | 42 (44%) | 11 (55%) | 0.23 |
|  |  | Septic Arthritis | 20 (21%) | 1 (5%) |  |
|  |  | Both | 33 (35%) | 8 (40%) |  |
|  | Concomitant syndrome (n, %) | Bacteremia without focus | 5 (5%) | 0 (0%) | 0.59 |
|  |  | Genitourinary | 10 (11%) | 3 (15%) | 0.70 |
|  |  | Pneumonia | 53 (56%) | 10 (50%) | 0.81 |
|  |  | Skin Abscess | 11 (12%) | 2 (10%) | 1.00 |
|  |  | Soft Tissue Abscess | 19 (20%) | 2 (10%) | 0.52 |
|  |  | Neurological | 7 (7%) | 0 (0%) | 0.60 |
|  | Occupational Exposure | | 13 (14%) | 3 (15%) | 1.0 |
|  | Recreational Exposure | | 80 (84%) | 19 (95%) | 0.30 |
|  | Bacteremia | | 67 (70%) | 15 (75%) | 0.79 |
|  | Shock | | 23 (24%) | 4 (20%) | 0.78 |
|  | ICU Admission | | 31 (33%) | 7 (35%) | 1.00 |
|  | Long bone involvement | | 42 (44%) | 13 (65%) | 0.09 |
| Treatment data | IV Antibiotic duration (days) | | 52.7 (23.4) | 47.9 (23.2) | 0.41 |
|  | PO Antibiotic duration (days) | | 173.9 (76.8) | 71.6 (65.0) | <0.01 |
|  | IV Antibiotic non-compliance | | 3 (3%) | 4 (20%) | 0.02 |
|  | PO Antibiotic non-compliance | | 28 (31%) | 10 (53%) | 0.11 |
| Outcome data | Died | Acute Illness | 5 (5%) | 2 (10%) | 0.09 |
|  |  | Recurrent melioidosis | 0 (0%) | 1 (5%) |  |

Supplementary Table 4: Comparison of arthritis – osteomyelitis foci in patients with combined infections but no long bone involvement.

| Septic arthritis foci | Osteomyelitis foci 1 | Osteomyelitis foci 2 |
| --- | --- | --- |
| Hand | Metacarpals / phalanges | |
| Midfoot / Toes | Metatarsals / phalanges | Other tarsal |
| Midfoot / Toes | Metatarsals / phalanges | |
| Knee | Metatarsals / phalanges | |
| Knee | Patella | |
| Ankle | Other tarsal |  |
| Ankle | Talus |  |
| Hip | Pelvis |  |
| Costovertebral / Sternoclavicular | Vertebrae | Rib / Sternum |

Supplementary Table 5: Comparison of predominant intravenous therapy and selected variables.

|  |  | Meropenem (n=33) | Ceftazidime (n=81) | Other (n=1) | p-value |
| --- | --- | --- | --- | --- | --- |
| Median clinical syndromes (IQR) | | 2.0 (2.0-3.0) | 2.0 (2.0-3.0) | 2.0 (2.0-2.0) | 0.61 |
| Bacteremia | | 27 (82%) | 54 (67%) | 1 (100%) | 0.19 |
| Recurrent  melioidosis | Recrudescence | 6 (18%) | 12 (15%) | 0 (0%) | <0.01 |
|  | Relapse | 1 (3%) | 1 (1%) | 0 (0%) |  |
| IV Antibiotic duration (days, CI) | | 49.2 (40.9-57.4) | 53.5 (48.5-58.6) | 3 (.) | 0.07 |
| Admission duration (days, CI) | | 33.9 (25.4-42.4) | 32.0 (26.8-37.3) | 3 (.) | 0.44 |
| Died due to Melioidosis | | 4 (12%) | 3 (4%) | 1 (100%) | <0.01 |
| IQR – Interquartile range; IV – Intravenous; CI – 95% Confidence interval | | | | | |

Supplementary Table 6: Comparison of predominant oral therapy and selected variables.

|  | | TMP/SMX (n=73) | Doxycycline (n=34) | Other (n=1) | p-value |
| --- | --- | --- | --- | --- | --- |
| Median clinical syndromes (IQR) | | 2.0 (2.0-3.0) | 2.0 (2.0-3.0) | 2.0 (2.0-2.0) | 0.73 |
| Bacteremia | | 49 (67%) | 27 (78%) | 0 (0%) | 0.13 |
| Recurrent  melioidosis | Recrudescence | 12 (16%) | 4 (12%) | 0 (0%) |  |
|  | Relapse | 0 (0%) | 1 (3%) | 1 (100%) |  |
| Oral Antibiotic duration (days, CI) | | 159.1 (140.0-178.2) | 155.1 (124.4-185.9) | 47 (.) | 0.41 |
| Admission duration (days, CI) | | 33.7 (28.1-39.4) | 31.1 (23.7-38.6) | 92 (.) | 0.04 |
| Died due to melioidosis | | 3 (4%) | 0 (0%) | 0 (0%) | 0.48 |
| TMP/SMX – Trimethoprim/Sulfamethoxazole; IQR – Interquartile range; CI – 95% Confidence interval | | | | | |

Supplementary Table 7: Summary of complications attributed to various therapeutics.

|  | Oral Agents | | | IV agents |  |
| --- | --- | --- | --- | --- | --- |
| Complication | TMP/SMX | Other | Ceftazidime | | Total |
| Cytopenia | 8 | 1 | 2 | | 11 |
| DRESS | 1 | 0 | 2 | | 3 |
| Gastrointestinal Upset | 2 | 1 | 0 | | 3 |
| Hepatitis | 3 | 0 | 1 | | 4 |
| Not specified | 3 | 0 | 2 | | 5 |
| Other | 1 | 5 | 0 | | 6 |
| Rash | 1 | 3 | 2 | | 6 |
| Renal dysfunction | 3 | 0 | 0 | | 3 |
| PICC ass. thrombi | NA | NA | 1 | | 1 |
| **Total** | 22 | 10 | 10 | |  |
| IV – Intravenous; TMP/SMX – Trimethoprim/Sulfamethoxazole; DRESS – Drug reaction with eosinophilia and systemic symptoms; PICC – Peripherally inserted central venous catheter; ass. – associated | | | | | |
